# Does short message service improve focused antenatal care visit and skilled birth attendance? A systematic review and meta-analysis of randomized clinical trials

**DOI:** 10.1186/s12978-018-0635-z

**Published:** 2018-11-22

**Authors:** Fasil Wagnew, Getenet Dessie, Animut Alebel, Henok Mulugeta, Yihalem Abebe Belay, Amanuel Alemu Abajobir

**Affiliations:** 1 College of health sciences, Debre Markos University, Debre Markos, Ethiopia; 2 College of health sciences, Bahir Dar University, Bahir Dar, Ethiopia; 3 Faculty of Medicine, the university of Queensland, Brisbane, Australia

**Keywords:** *MHealth*, Phone text messaging, FANC, LMICs, Systematic review and meta-analysis

## Abstract

**Background:**

In low resource circumstances, non-adherence for available health services is a major cause of inefficiency in health care delivery. MHealth has been projected as a possible solution to support women during pregnancy, birth and puerperium period, to increase the uptake of essential maternal services.

**Objectives:**

This systematic review and meta-analysis study was aimed to determine the effectiveness of short message services (SMS)on Focused Antenatal Care (FANC) visits and the attendance of skilled birth professionals in Low and Middle Income Countries (LMICs).

**Methods:**

We searched a broad body of literature from electronic databases–Cochrane review, CINAHL, PsycINFO, PubMed and Google Scholar to collect comprehensive evidence on the role of SMS on FANC visits and skilled birth attendance. We extracted data from randomized clinical trials (RCTs) only. Meta-analyses were conducted using random-effects models with inverse variance method in Review Manager (RevMan) computer software. Qualities of the included studies were determined by GRADEpro, and risk of bias was assessed using Cochrane Collaboration risk of bias tool.

**Results:**

Of the 1224 non-duplicated articles screened, only 7 RCT studies representing 8324 participants met eligibility criteria and included in this synthesis. On aggregate, there were statistically significant associations in experimental group in that pregnant mothers who received text messaging had a 174% increase in FANC visits (OR = 2.74 (95% CI: 1.41, 5.32) and 82% in skilled birth attendance (OR = 1.82 (95% CI; 1.33, 2.49). The I^2^ test result indicated high heterogeneity I^2^ = 78% (*P* < .001). The overall qualities of included studies were moderate, and had low risk of bias.

**Conclusions:**

SMS has positive effects for the uptake of FANC visits and skilled birth attendance in LMICs. A short messaging service targeting pregnant woman is an invaluable, affordable intervention to improve maternal healthcare seeking behaviors.

**Electronic supplementary material:**

The online version of this article (10.1186/s12978-018-0635-z) contains supplementary material, which is available to authorized users.

## Plain English summary

Non-attendance of available services is a major cause of inefficiency in healthcare delivery. *MHealth* has been projected as a possible solution to support women during pregnancy, birth and puerperium period, to increase the uptake of essential maternal services. This study was determine the impact of SMS on FANC visits and the attendance of skilled birth professionals in LMICs.

A broad range of databases published between 2008 and 2017– Cochrane, CINAHL, PsycINFO, PubMed, Web of Science and Google scholar–were used to search relevant literature**.** Based on a priori set criteria, only 7 RCTs relevant to this study were systematically reviewed. Two reviewers extracted the required information from the relevant articles separately. Meta-analyses were conducted using random-effects models with inverse variance method in Review Manager (RevMan) computer software.

On aggregate, there were statistically significant associations in experimental groups in that pregnant mothers who received text messaging had a 174% increase in FANC visits (OR = 2.74 (95% CI: 1.41, 5.32) and 82% in skilled birth attendance (OR = 1.82 (95% CI: 1.33, 2.49). The overall qualities of included studies were moderate, and had low risk of bias.

In conclusion; SMS has positive effects for the uptake of FANC visits and skilled birth attendance in LMICs.

## Background

Despite ongoing efforts to improve maternal and child health in developing countries, mortality rates still remain high, with 1 in 160 lifetime risk of maternal mortality in developing regions as compared to 1 in 3700 for women living in developed regions [1]. Limited access to preventive maternal health services, poor administration, limited logistic and technical ability, insufficient financial assets and scarcity of skilled health personnel’s are some of the reasons for this disparity [2].

The essential interventions with proven role to reduce maternal mortality include antenatal care (ANC) (during pregnancy), skilled birth attendances (SBA) during intra-partum (labour and delivery) access to contraception for postponing, spacing and limiting because the worldwide increase in postponing, spacing and limiting pregnancies has, in itself, been the most important single factor for dramatically reducing the mortality of mothers and their children the last 50 years [3, 4]. Babies do much better when they are well-spaced [4]. In Ethiopia, infants born less than two years after a previous birth have particularly high under-five mortality rates (179 deaths per 1000 live births, compared with 72 deaths per 1000 live births for infants born three years after the previous birth). Twenty percent of infants in Ethiopia are born less than two years after a previous birth [5] and the postpartum periods (follow-up after delivery) [6]. Indeed, studies in Tanzania and Ethiopia have confirmed the capacity of focused ANC (FANC) and postnatal care (PNC) provision to mitigate maternal mortality [7–9]. Data from Demographic and Health Surveys (DHS), however, reported that two-thirds of women deliver without skilled birth attendance, only 13% having received a postnatal check-up within 48 h in 23 African countries [10]. In addition, about half of pregnant, in low and middle-income countries (LMICs), attain the World Health Organization (WHO) recommended level of at least four ANC visits (i.e., FANC) [11]. Nonetheless, non-attendance of services is a major cause of inefficiency in healthcare delivery. The field of mHealth, or mobile health, has been projected as a possible solution to many of the problems in LMICs in tackling workforce scarcity and health education opacity, as well as, *mHealth* has been promoted for record keeping or data recording in general [12].

On the other hand, there is a rapid increase in mobile phone coverage in developing countries bringing up a new unprecedented opportunities for providing health information to a large number of people at a low price [13–15]. Radio broadcasting is not limited to a target group. The international telecommunication union reported that in 2013, global mobile-phone subscriptions reached 6.8 billion and that the mobile-cellular penetration rate or the number of active mobile phone users within a specific population reached 89% in developing countries [16]. An estimated 184 million women own mobile phones in low-income countries [17]. More than 60% of individuals now have access to a mobile phone in sub-Saharan Africa [18].

One of the vital areas tackled by *mHealth* interventions is the support provided to women during pregnancy, birth and puerperium to reduce maternal and child mortality [19]. Prior studies including one systematic review have revealed that text messaging may be a capable and effective tool to provide support, offer messages and encourage visits of women during prenatal and postpartum periods [20–23]. Intervention by mobile telephone, including contact by short message service and multimedia message service (MMS) pictures, deliver frequent reminders on nutritional and physical activities or recommendations.. These interventions are convenient and potentially cost-effective in encouraging pregnant women to maintain healthy behaviors. The use of mobile phone-based technology in healthcare has emerged to augment the healthcare services where the population is underserved, especially in rural areas [24]. During pregnancy, mHealth can be used for point-of-care remote consultation, facilitating referral and access to health facilities and for promoting timely contact (appointment) with the community health workers [16].

Nonetheless, to our knowledge, none of these studies explored a pooled effect of SMS on FANC visits and skilled birth attendance. Thus, the evidence base is still unclear, inconsistence and inconclusive. This systematic review and meta-analysis was aimed to determine the effectiveness of short message services on FANC visits and SBA rate in LMICs.

## Methods

### Search methods for identification of studies

A broad range of databases–Cochrane, CINAHL, PsycINFO, PubMed, Web of Science and Google scholar– published between 2008 and 2017 were used to search relevant literature**.** The search was extended to high quality studies by retrieving from the reference lists of included studies. The search strategy used the combination of the following key terms: “mHealth” “mobile phone*”,“SMS”, “text message”, “telemedicine*”, AND “pregnancy”, “maternal health”, “prenatal Care” AND “LMICs”. Searches were done by two reviewers (FW and GD) independently and any conflicts were resolved by discussion, and last author (AAA) was consulted whenever appropriate.. Search strategy are provided in Additional file 1. Boolean operators – ‘OR’ or ‘AND’ – were used*.* Endnote reference manager software was used to collect and organize search outcomes and for removal of duplicate articles. Search for the study was carried out from 1 February 2018 to 30 April 2018.

### Included studies

In order to reduce heterogeneity and increase comparability across included studies, we considered studies with only randomized clinical trial that determined the role of SMS for pregnant mothers.

Population pregnant women in LMICs who attended ANC visit(s) in all settings (i.e. primary care settings (services in primary health care), outpatient settings (outpatient clinics), community settings (public health services) and hospital settings).

### Types of interventions

Interventions that use SMS as reminders for a scheduled health appointment(s) were included. We excluded appointment reminders provided for other services, for example, for socialization purposes.

### Types of outcome measures

Primary outcome: the effect of mobile phone texting message service on FANC visits.

Secondary outcome: the effect of mobile phone texting message service on the skilled birth attendance. Primary and secondary outcomes were considered based on their natural order (i.e., FANC visits for pregnancy, and then for delivery).

### Data extraction

The data extraction format was constructed and pilot-tested with a subset of eligible studies, and then summarized using a table. Two reviewers (FW, GD) separately extracted the required information from the relevant articles. Further information was request from primary authors through email (whenever indicated). Discrepancies were resolved by consensus, whenever appropriate. Data extracted from the included studies: author’s name, year of publication, country of study, participant characteristics, study design, types of interventions and main findings (FANC and SBA). For dichotomous data, we extracted the number of participants with outcome of interest and total sample size.

### Quality of evidence

The overall quality of evidence was evaluated using the Grades of Recommendation, Assessment, Development and Evaluation (GRADE) methods [25]. GRADE profiler was used to compute the evidence profile and categorize the quality of evidence. The quality of evidence was classified as: (1) high quality (further research is extremely unlikely to change the credibility of the pooled results); (2) moderate quality (further research is likely to influence the credibility of pooled results and may change the estimate); (3) low quality (further research is extremely likely to influence the credibility of pooled results and likely to change the estimate); and (4) very low quality (the pooled results have extreme uncertainty) [25].

### Assessment of risk of bias

Two authors (FW, GD) evaluated the risk of bias of the included studies using Cochrane Collaboration tool. Methodological quality of each study was appraised by retrieving information on five components related to the design, execution and reporting of randomized trials: randomization technique, allocation concealment, blinding, manner of handling withdrawals and comparability of randomized groups, with respect to baseline characteristics [26]. Studies were considered to have a low risk of bias when all key aspects were assessed and found to be at low risk for bias [26]. Consistent discussion was in place to settle any controversial idea, or a third author (AAA) was used as a mediator.

### Data synthesis and analysis

Characteristics of the 7 included RCTs were summarized and presented in a descriptive table (Table 1). The extracted data were entered in to Microsoft excel spreadsheet and then exported to RevMan version 5.3 software for meta-analysis. Pooled effects odds ratio (OR) and its corresponding 95% confidence interval (CI) was estimated by using the inverse-variance method of random-effects model [27]. Funnel plot and egger test were used to test for publication bias. Heterogeneity between studies was assessed by calculating the I^2^ statistic and its corresponding 95% CI using Rev-Man version 5.3 [28]. To verify the results, two researchers (FW, GD) independently computed main statistical analysis and checked for consistency.

**Table 1:** Descriptive review of RCT relevant studies on the effect of SMSon pregnant women’s health care services uptake

Authors	Country	Participants	*Mhealth* Interventions		Outcome	Main finding
			Experimental group	Control Group		
Jareethum et al. 2008 [57]	Thailand	Pregnant women / Size: Intervention: 32, Control: 29	Text messaging: twice weekly	routine ANC and advice	Maternal satisfaction	Satisfaction scores of antenatal and perinatal periods were significantly higher in the study group compared to the control
Lund et al.,2012 [55]	Zanzibar:	Intervension;1311 Control;1239	Mobile phone text-message, twice a week	routine ANC and advice	Primary outcome: skilled attendance at delivery	The mobile phone intervention significantly Increased skilled delivery attendance among pregnant women (OR: 1.69(1.44–1.98)).
T.Fedha,2014 [64]	Kenya	Intervension:191 Control:206	reminded every fortnightly of the next visit to the clinic and given advice on pregnancy updates and advice	Allowed to continue with routine clinics with no mobile advice or updates support	Primary outcome: FANC Secondary outcome: skilled birth attendance	mobile telephone service for pregnant mother enhance maternal health care ANC visits (OR: 2.89(1.51–5.53))and skilled birth attendance(OR: 2.73(1.60–4.65))
Lau et al. 2014 [59]	South Africa	Size: Intervention: 102, Control: 104	Text messaging: staggered according to the week of pregnancy	routine ANC and advice	To compare the control and intervention group’s knowledge	No statistically significant difference in score in any of the 9 questions between the intervention and control
Lund et al.,2014 [56]	Zanzibar:	Intervension;1311 Control;1239	Mobile phone text-message	routine ANC and advice	Primary outcome: FANC visits -secondary outcome: tetanus vaccination, other preventive services for malaria, etc.	In the Intervention group 44% of the women received four or more antenatal care visits versus 31% in the control group.
Atnafu, 2017 [58]	Ethiopia	Size; Intervention: 1080, Control: 1080	SMS based mobile phone intervention in most of the selected MCH service indicators	No SMS	role of mobile phone SMS MCH outcomes	The proportion of mothers receiving more than four ANC visits increased significantly (increased from 5.21% at baseline to 29.75%).
*Bangal VB* et al. *2017* [54]	India	Intervention: 200 Control: 200	Mobile phone calls, as reminders about next visit and text messages (SMS)	Control group: women received routine ANC and advice as per hospital protocol.	percentage of pregnant women coming for FANC, percentage of institutional delivery and postnatal check-ups.	Mobile phone intervention significantly increased the percentage of women receiving the recommended four antenatal visits (OR:4.4(2.86–6.77)) and non-significant effect on SBA (OR: 1.33(0.68–259)).

### Operational definition

**Focused anti-natal care:** recommends that all health pregnant women should have a minimum of four scheduled comprehensive antenatal visits during pregnancy.

**MHealth:** use of mobile and wireless technologies to support the achievement of health objectives.

**Skilled birth attendants** are midwifes, doctors or nurses who have been educated and trained in the skills needed to manage pregnancies, childbirth and the immediate postnatal period, including the identification, management and referral of complications in women and newborns.

## Results

### Study inclusion

The search strategy identified 1453 articles. Of these, 229 articles were excluded due to duplication. A total of 1224 unique citations met potential inclusion for this review. The defined inclusion criteria were applied to the title, and where necessary, abstracts for these citations were revised by two researchers (FW, GD), independently. The reviewers agreed that 33 citations met inclusion criteria and a further 1191 citations did not meet inclusion criteria for the review.

**Fig. 1:**
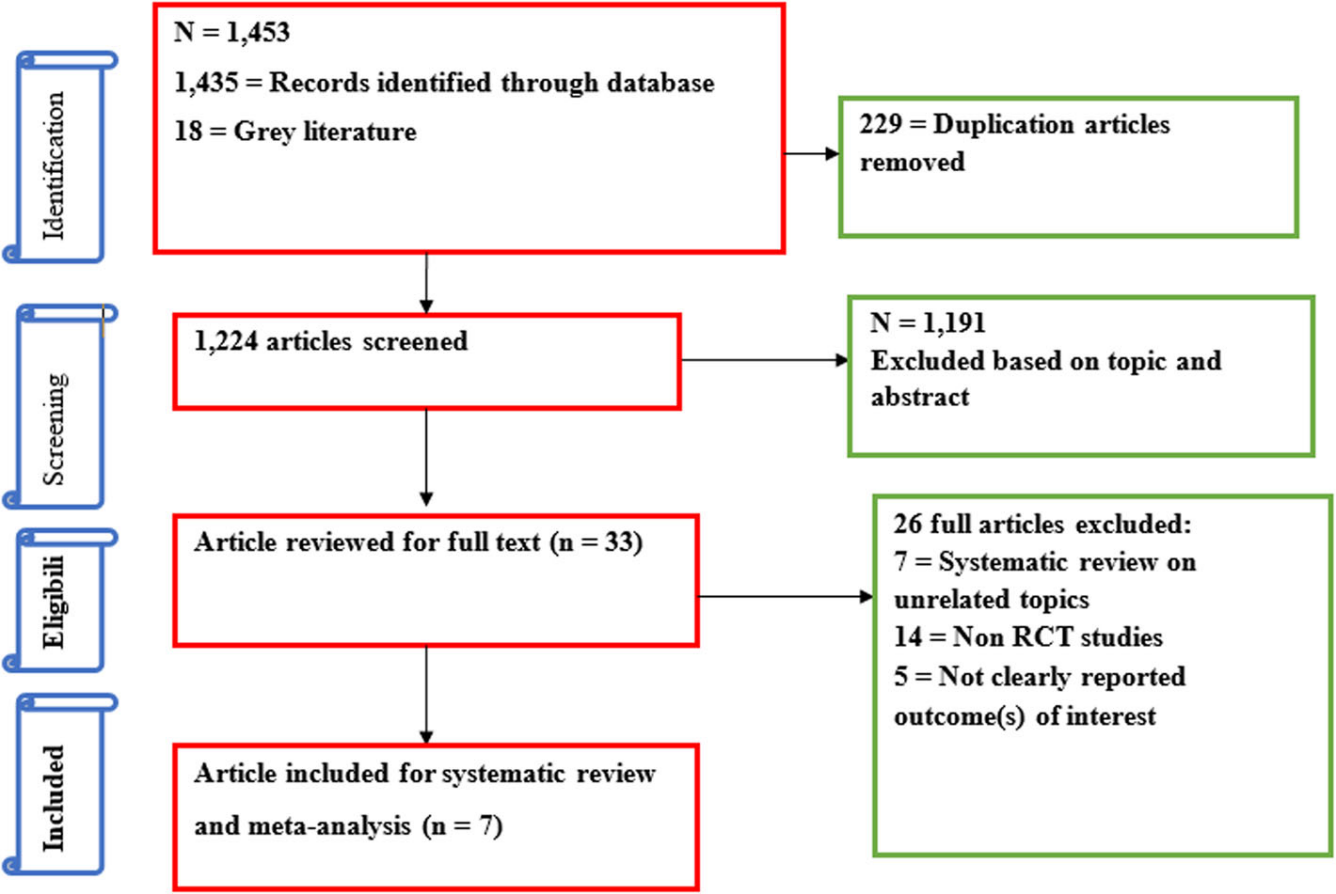
Flow chart describing selection of studies for a systematic review and meta-analysis of the effect of SMSon FANC visits and skilled birth attendance among pregnant women in LMICs

Thirty-three full text articles were further screened. Of these, 26 were excluded since 7 articles were systematic reviews on other related topics [16, 29–34], 14 were non-controlled clinical trials [35–48], and 5 RCTs reported different outcomes of interest [49–53]. Based on a priori set criteria, only 7 RCTs relevant to this study were systematically reviewed (Fig. 1). A meta-analysis was done from 4RCTs that specifically determined the effect of SMS on FANC visits and skilled birth attendance. (Table 1).

### Characteristics of the included studies

RCTs at clinic- and community-levels on SMS for pregnant mothers were included. Studies those explicitly addressed the effect of *mHealth* interventions on FANC visits and skilled birth attendance, and reported that the mobile phone interventions increase FANC visits (> 4 visits) and skilled birth attendance at delivery were conducted in India [54], Zanzibar [55, 56] and Kenya [27]. Study done by **Atnafu**, a community-based RCT, reported that the proportion of mothers receiving SMS were more likely to receive FANC as compared to no interventions group. The number of study participants (intervention group) ranged from 32 [57] to 1311 [55]. Study characteristics and primary outcomes of reviewed articles were summarized in Table1. Finally, a meta-analysis was done using 3 RCTs studies.

### Pooled effect of SMS on FANC and SBA

**Fig. 2:**
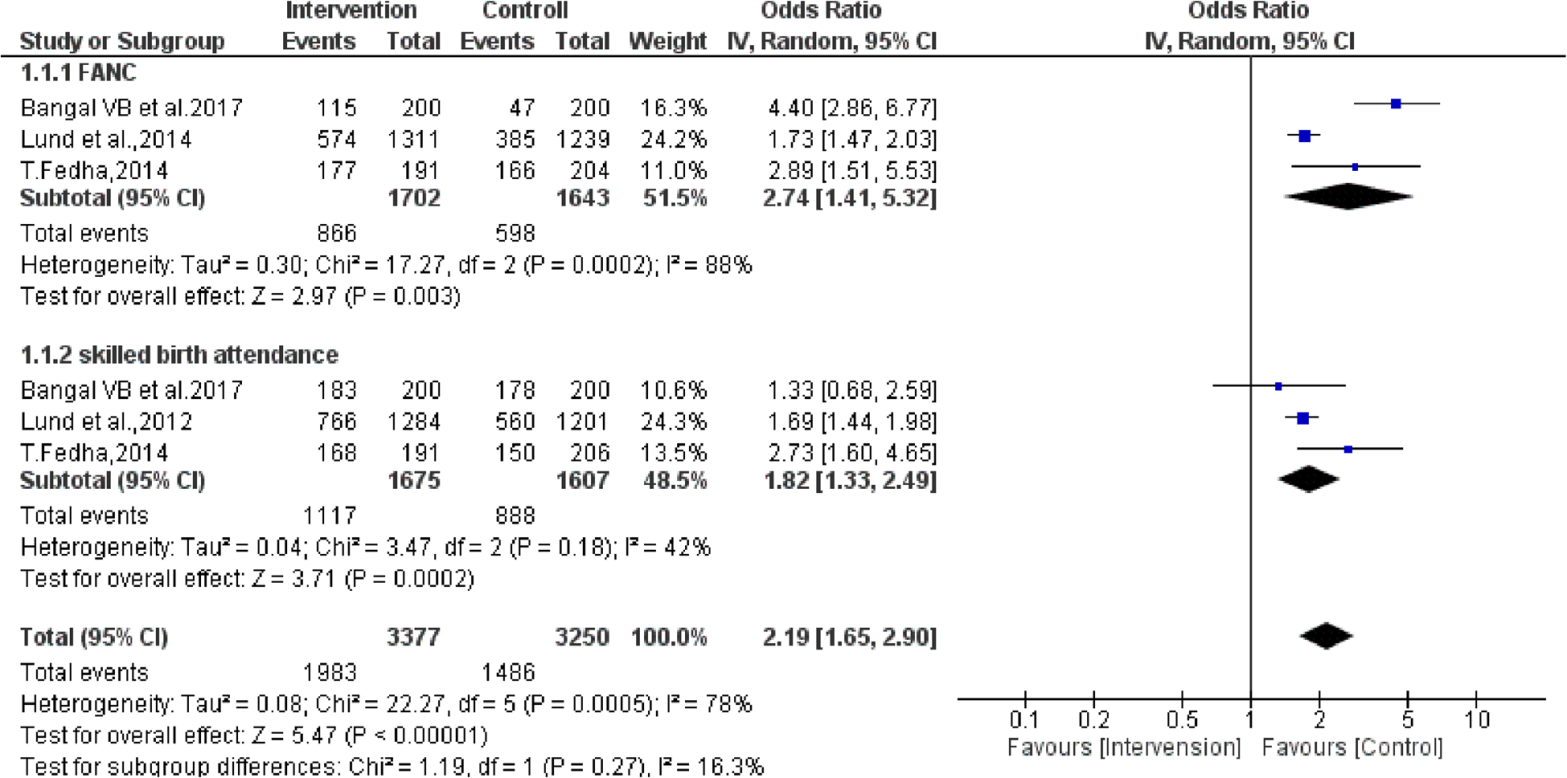
Forest plot of the 7 RCT studies that quantitatively assessed the effect of mobile phone messaging on maternal healthcare services uptake during pregnancy and at birth

**Fig. 3:**
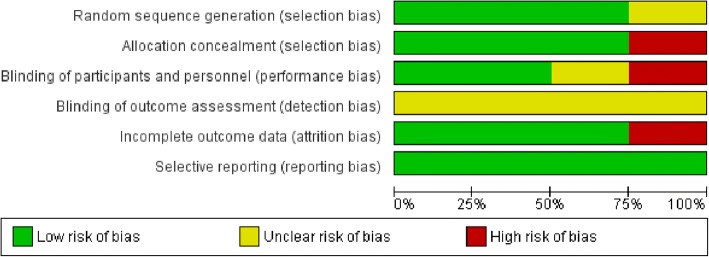
Risk of bias graph: review authors’ judgments about each risk of bias item presented as percentages across all included studies

**Fig. 4:**
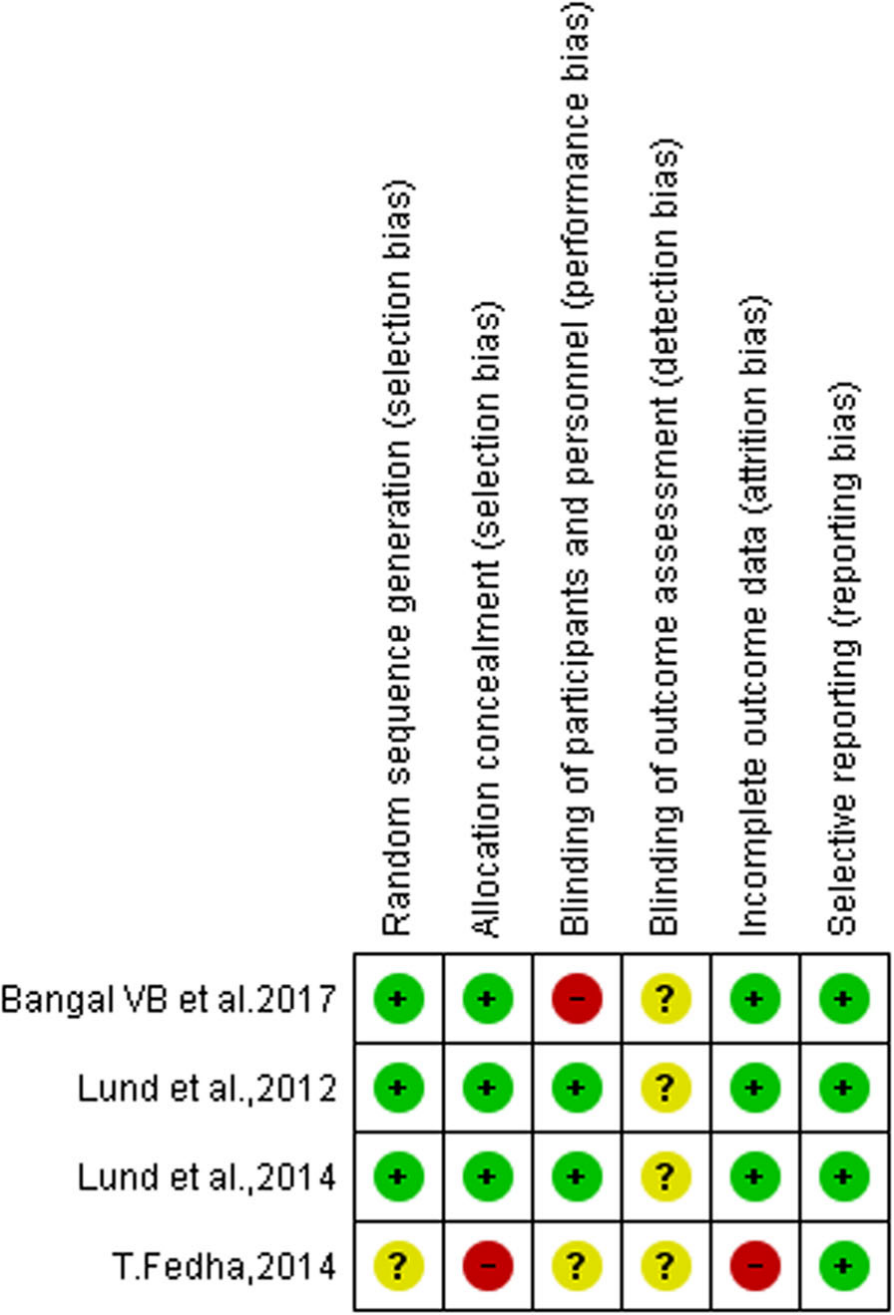
Risk of bias summary: review authors’ judgments about each risk of bias item for each included study

Three studies [57–59] were not included in meta-analysis because of inconsistent results. Two studies [57, 58] supported for maternal care delivery system which reported that mHealth intervention seems to have positive impact on FANC and maternal satisfaction. However, study done by Lau et al., 2014 [59] showed that SMS had not significant effect on FANC for both the intervention and experimental groups. Three out of the 7 included studies assessed the impact of SMS on FANC visits (as a primary outcome), and the other 3 included studies assessed the impact of SMS on SBA at delivery (as a secondary outcome). A total of 3345 participants were included in the meta-analysis (Fig. 2). On aggregate, there were statistically significant associations in experimental groups in that pregnant mothers who received text messaging had a 174% increase in FANC visits (OR = 2.74 (95% CI: 1.41, 5.32) and 82% in skilled birth attendance (OR = 1.82 (95% CI: 1.33, 2.49). The I^2^ test result indicated high heterogeneity (*P* < .001). The overall qualities of included studies had low risk of bias (Figs. 3 and 4). As Figs. 3 and 4 display a graphical explanation of the risk of bias across the studies using the Cochrane Collaboration tool. All of the studies adequately described how the randomized allocation sequence was generated, and all but one [60] of the studies fully concealed the allocation prior to assignment and not exper from attrition bias generated from incomplete outcome data. There are no studies clearly reported blinding of outcome assessment (detection bias). Evidence on the level of quality was evaluated by using GRADE pro criteria, which gave as a moderate level of quality (Table 2).

**Table 2:** GRADEpro level of quality evidences assessment

Certainty assessment							№ of patients		Effect		Certainty
№ of studies	Study design	Risk of bias	Inconsistency	Indirectness	Imprecision	Other considerations	phone text messaging	no interventions	Relative (95% CI)	Absolute (95% CI)	
FANC visit (assessed with: OR)											
3	Randomized trials	not serious	serious	not serious	not serious	none	866/1702 (50.9%)	598/1643 (36.4%)	OR --(1.41 to 5.35)	-- per 1000 (from 83 more to 390 more)	⨁⨁⨁◯ MODERATE
Skilled birth Attendance(assessed with: OR)											
3	Randomized trials	not serious	serious	not serious	not serious	none	947/1675 (56.5%)	731/1607 (45.5%)	OR -- (0.80 to 2.78)	-- per 1000 (from 55 fewer to 244 more)	⨁⨁⨁◯ MODERATE

*CI* Confidence interval; *OR* Odds ratio

## Discussion

### Principal findings

This meta-analysis detected a statistically significant increase in FANC visits among pregnant mothers who had received text messages as compared to their counterparts. This study also found a significant difference between intervention and control groups of pregnant mothers who received text messaging and the likelihood of having their delivery attended by skilled health personnel as compared to those who did not receive text messaging.

this finding is in keeping with a systematic review done in Ethiopia that also showed that *mHealth* tools are effective to influencing maternal and child health services utilization by enhancing ANC/PNC attendances and delivery in health institutions [29]. Another systematic review done by Feroz (2017) [31] that reported *mHealth* interventions, particularly those delivered through SMS, were associated with improved utilization of preventive maternal healthcare services including uptake of recommended ANC and PNC services. This study also identified a significant difference between intervention and control groups of pregnant mothers who received text messaging and the likelihood of having their delivery attended by skilled health personnel as compared to those who did not receive text messaging. Consistently, a systematic review done by Colaci (2017) reported that phone text offered an opportunity to enhance acceptability of prenatal and obstetric care including skilled birth attendance [30]. This is because SMS interventions (e.g., reminders, feedback, etc.) boosts self-efficacy, enhances provision of social support and create peer-to-peer networks. It may also improve health-seeking behaviors [60, 61]. Furthermore, Mobile phone services, especially in certain population groups such as teenage girls and pregnant women in remote areas, have facilitated access to some healthcare services. Therefore, SMS messaging has been used as an appointment reminder and can provide basic health information, notably throughout pregnancy period [62]. Newer and more cost-effective systems are being sought. This technology can also cost effectively be adapted to address the basic health needs of those living in remote and rural areas [63].

Although few studies [57–59] were not included in the meta-analysis because of unclear evidence and inconsistent results, these studies strongly supported that *mHealth* intervention can enhance client behavioral change and mental satisfaction. This could increase the uptake of maternal healthcare services such as ANC, SBA at delivery and PNC.

Interestingly, the quality of evidence was moderate, suggesting the observed effect was close to true effect, and that there was non-significant publication bias. The present study differs from the previous studies in that the eligiblility criteria were more rigorous, including only on RCTs, a meta-analysis, and included a quality assessment of the included articles.

The findings of this meta-analysis must be interpreted cautiously in view of the strengths and limitations of the included trials. To our knowledge, this is the first systematic review protocol with innovative approach including pregnancy that will attempt to assess the pooled effect of mobile text messaging on promoting FANC and SBA. As well, this study is the fact that studies included in this meta-analysis were well-performed and high-quality RCTs. Additionally, with the enlarged size of the participants, we have enhanced the statistical power to provide more precise and reliable effect estimates. Nonetheless, some of important limitations included the inclusion of studies published only in English (language bias) may compromise representativeness. This study represented only studies reported from six Countries, which may reflect selection bias due to the limited number of studies included from other equivalent countries.

### Implications for research

Application of the ‘right’ strategy and/or technology is an essential component of developing evidence-based practice and ultimately improving maternal health care. It is reasonable that in a resource-constrained setting, mHealth interventions should be implemented in the health care system to reduce maternal and child mortality. This systematic review and meta-analysis has pivotal role in strengthening the capacity of institutions to make evidence-based decisions through enhancing the application of mHealth. In conclusion, this findings have broad implications for public health policy in designing and implementing mHealth interventions in low-resource settings around the world. Furthermore, this study will help inform clinical practice and future studies on the effectiveness of media platforms.

## Conclusions

SMS seem to have positive effects for the uptake of FANC visits and SBA in LMICs. Thus, mobile phone applications may contribute towards improved maternal healthcare seeking behavior and should be considered by public *health* leaders and *policy makers* in resource-limited settings.

## Additional file

Additional file 1: Search strategy. (DOCX 13 kb)

## Abbreviations

FANC: Focused Antenatal Care visits
GRADE: Grades of Recommendation, Assessment, Development and Evaluation
LMICs: Low and Middle Income Countries
MMS: Multimedia Message Service
OR: Odds Ratio
PNC: Postnatal Care
RCT: Randomized Clinical Trial
SBA: Skilled Birth Attendance
SMS: Short Message Service
WHO: World Health Organization
