# Supplementary material for: Does short message service improve focused antenatal care visit and skilled birth attendance? A systematic review and meta-analysis of randomized clinical trials
Source: Reprod Health. 2018 Nov 22;15:191. doi: 10.1186/s12978-018-0635-z (PMC6249748; doi:10.1186/s12978-018-0635-z)
Supplement: Supplementary file 1 — Search strategy. (DOCX 13 kb) [file 12978_2018_635_MOESM1_ESM.docx]

| **Databases** | **Search Terms** |
| --- | --- |
| \| **PubMed** \| \| --- \| | \| **MeSH terms:** mHealth (MeSH), short message service (MeSH), cellular phone (MeSH), telemedicine (MeSH), SMS(MeSH), pregnancy (MeSH), perinatal care (MeSH).  **Search Terms:** (mobile messag* OR short message service* OR SMS* OR tele medicine* OR texting [tw]) AND (pregnancy OR perinatal care[MeSH] AND low-and middle-income countries OR LMICs AND English [la] \| \| --- \| |
| \| **CINAHL (Plus with full text)** \| \| --- \| | \| **Thesaurus terms:** text messaging, pregnancy, low or middle-income countries  **Search Terms:**  "mobile phon*" OR telemedicine* OR "cellular phon*" OR "text messag*" OR "short message service" AND pregnancy  Low-income countries OR middle-income countries OR LMICs  (S1): filter: English, peer reviewed, exclude Medline  (S2): with additional filter: RCT, randomized controlled trial  (S3): with additional filter: LMICs, low-and middle-income countries  S1 OR S2 OR S3 \| \| --- \| |
| \| **Cochrane Library** \| \| --- \| | \| **MeSH terms:** mHealth (MeSH), short message service (MeSH), cellular phone (MeSH), telemedicine (MeSH), SMS (MeSH), pregnancy (MeSH).  **Search Terms:** (mobile messag* OR short message service* OR SMS* OR tele medicine* OR texting [tw]) AND (pregnancy[MeSH] OR perinatal care[MeSH] AND low-and middle-income countries OR LMICs AND English [la] \| \| --- \| |
| \| **PsycINFO** \| \| --- \| | \| **Thesaurus terms:** cellular phone, messages, telemedicine, pregnancy, perinatal care, low-and middle-income countries  **Search Terms:**  "mhealth*" OR short message service* OR "SMS" OR "text messag*" OR "telemedicine" AND pregnancy OR perinatal* AND low-and middle income countries  filter: English, peer reviewed \| \| --- \| |
| \| **Embase** \| \| --- \| | \| **Emtree terms:** text messaging, mhealth, pregnancy  **Search 1 Terms:** (mhealth* OR sort message service OR text messag* OR telemedicine OR texting) AND (pregnancy OR perinatal* AND RCT OR randomized clinical trial AND (embase) NOT (medline)  **Search 2 Terms:**  (mhealth NEXT/1 phone* OR short messag* OR SMS NEXT/1 phone* OR telemedicine NEXT/1 phone* OR text NEXT/1 messag* OR short NEXT/1 messag* OR 'short message service' OR texting) AND (pregnancy OR perinatal care OR randomized clinical trial OR 'RCT') AND english:la AND [embase]/lim NOT [medline]/lim  filter English only and excluded Medline \| \| --- \| |
| **Web of Science** | **Search Terms:** "mhealth*" OR smartphon* OR "cellular phon*" OR "text messag*" OR "short message service" OR texting AND  Pregnancy OR perinatal care AND "RCT" OR randomized clinical trial  Filter English only.  Included Science Citation Index Expanded and Social Sciences Citation Index &  Excluded Arts and Humanities Citation Index |
| **Grey Literature; Google and Google Scholar** | Variety of key terms used from above searches |
